# Standard Reference Specimens in Quality Control of Engineering Surfaces

**DOI:** 10.6028/jres.096.015

**Published:** 1991

**Authors:** J. F. Song, T. V. Vorburger

**Affiliations:** National Institute of Standards and Technology, Gaithersburg, MD 20899

**Keywords:** calibration, ISO, quality control, roughness, roughness average, specimen, SRM, standard, standard reference material, step height, stylus, surface

## Abstract

In the quality control of engineering surfaces, we aim to understand and maintain a good relationship between the manufacturing process and surface function. This is achieved by controlling the surface texture. The control process involves: 1) learning the functional parameters and their control values through controlled experiments or through a long history of production and use; 2) maintaining high accuracy and reproducibility with measurements not only of roughness calibration specimens but also of real engineering parts. In this paper, the characteristics, utilizations, and limitations of different classes of precision roughness calibration specimens are described. A measuring procedure of engineering surfaces, based on the calibration procedure of roughness specimens at NIST, is proposed. This procedure involves utilization of check specimens with waveform, wavelength, and other roughness parameters similar to functioning engineering surfaces. These check specimens would be certified under standardized reference measuring conditions, or by a reference instrument, and could be used for overall checking of the measuring procedure and for maintaining accuracy and agreement in engineering surface measurement. The concept of “surface texture design” is also suggested, which involves designing the engineering surface texture, the manufacturing process, and the quality control procedure to meet the optimal functional needs.

## 1. Introduction

### 1.1 Review of Quality Control for Engineering Surfaces

At the turn of this century, it became apparent that the surfaces of mechanical parts could be an important factor in determining how well the parts functioned. This seemed most true in cases where two components were in static or dynamic contact. Even earlier came the realization that no function of a part could be guaranteed unless the method of manufacture could be controlled. The control was usually achieved in those days by including many details of the manufacturing process, such as “rough machining,” “medium machining,” and “fine machining,” or equivalent symbols on the engineering drawing. By carefully following the procedure laid down, some degree of control of the manufacture, and hence the function, could be achieved [1,2].

This early engineering surface quality control involved a control loop consisting of surface manufacture, surface texture, and surface function. The control process depended on the limitations of the machining process involved, as well as the arbitrary opinions of operator and inspector which, all too often, did not coincide. The resultant problems became increasingly acute as the demand for more comprehensive specifications increased to keep pace with technological development [2].

It was not until the early 1930s, however, that any serious attempt was made to quantify the magnitude and nature of the relationship between surface texture and function [1]. The stylus instrument, a technology now more than half a century old [3], laid the ground work in quantifying the surface texture, and made engineering surface quality control possible by enabling the measurement and control of the values of various surface parameters. Both the manufacturer and the consumer benefited from this control. One of the examples was from the automobile industry. Automobiles no longer require running-in, their engines and transmissions are reliable and long lived, and their fuel economy has been improved appreciably by reduced internal friction [4].

Digital methods and computer techniques have further revolutionized surface metrology because in principle, a digitizing surface instrument with a digital computer is infinitely flexible and can produce almost any analysis of the surface geometry that is required [5]. As a result, there has been a significant increase in standards, definitions, algorithms and parameters (dozens of them) which, on the one hand, describe the surface geometry from different views, but, on the other hand, force the engineer to cope with a variety of national and international standards whose interrelationships are obscure [6].

Meanwhile, other surface measurement techniques based on different principles, such as optical interference [7–8], optical scattering, [8–10], capacitance [11], and ultrasound [12] were developed to detect the surface texture. These techniques provided more means of surface measurements for industry, such as on-line, area-based scattering measurement [13] and non-contact, optical profiling and mapping [14–16]. Many users of these new instruments and techniques attempt traceability or comparison to the stylus instrument measurements [10–12].

The field of surface profiling has been expanded to the atomic scale in recent years by the development of the scanning tunneling microscope (STM) [17–21] and its offshoot, the atomic force microscope (AFM) [21–24]. Long trace STM has also been developed now for the smooth engineering surface measurement [25].

All of these instruments and techniques now reaching the market or still in the development stage are complicating the situation further. When these instruments, all of them scientifically based, well designed, and equipped with modern digital and computer techniques, get together to measure the same surface, a considerable disagreement, say 50% or more, [26] could be obtained in the measured parameters.

This divergence of methods should not be a surprise to the instrument manufacturer, since surface texture measurement results not only provide an assessment of the surface under examination, but also an assessment of the instrument used in that examination. In many cases, differences between measurement results from different instruments are not due to the fact that one surface measuring instrument is right and the other wrong. A difference is more likely due to the different measurement strategies and internal variables that have been adopted for each instrument. In the case of stylus instruments, a few of these considerations include stylus size, instrument bandwidth, computational algorithms [5], and reference datums.

Also, these differences should not be a surprise to a mechanical engineer, since the so called “same surface” is actually not the same everywhere. Even on the surface of calibration specimens, a few percent variation of their roughness average (*R*_a_) [27] value, or even more than 15% on other parameters has been observed [28,29]. On real engineering surfaces, 50% or more variations of their surface parameter values is commonplace [29]. And it is almost impossible to make an engineering surface with uniformity in surface texture less than 1% [30]. It has also been known to aviation engineers that, during the aircraft engine manufacturing process, the surface roughness of the parts in the continuous grinding process always changed more than ±50% in a working day, but that a variation of 30% in the surface texture usually had only a very small effect on the parts’ function [30].

However, such differences are difficult for an inspector to accept. The inspector would base his judgments of “pass” or “reject” on the parameter values of his surface texture measurements. His consideration of the accuracy and agreement of his measurements could be much more rigorous than the requirements of surface function. And differences of opinion between manufacturers and their customers over the functional suitability of components have occurred when parameter values were out of their specified tolerances on the customer’s instrument but not on the manufacturer’s [5].

One of the considerations in quality control is to assign a “safety margin,” like that used in size tolerance quality control. The measured roughness value would have to be smaller than the designed tolerance for maximum roughness because of the uncertainty of the measurement. However, this is not a good solution to our problem. By no means does the smoother surface necessarily produce the better function. Some surface functions, such as lubrication, are degraded when the surface is overfinished. Furthermore, the costs of finishing products increase rapidly as the *R*_a_ values of the surface finish decrease, especially on smooth engineering surfaces [2,27,31].

The fractional uncertainty of surface roughness measurement increases rapidly as its *R*_a_ value decreases. However, surface quality control is most important on the surfaces of critical parts having small size tolerance and fine surface structure to meet some particular functional [32] requirements. The peak-to-valley roughness (*R*_t_ or *R*_max_) usually takes up a significant fraction of the size tolerance, for example [33], between 31% and 49% of tolerance values for different diameter ranges and tolerance grades. That fraction increases as the size or tolerance of the workpiece decreases. Due to the developments in the automobile, aviation, space, and microelectronics industries, many critical parts with small size tolerance and fine surface finish have appeared. These smooth engineering surfaces need a high degree of quality control in their manufacturing process for their proper function during use. At present, however, surface metrology cannot meet these needs very well. Therefore, more and more people are aware of the importance of accuracy and agreement in their engineering surface measurements, especially of smooth engineering surfaces. As a representative of one automobile manufacturer said, when he visited our lab about a year ago, “We do pay money for our (surface) measuring error.”

### 1.2 Outlook for Quality Control of Engineering Surfaces

The solution to the problems of engineering surface quality control involves two aspects:

1. To recognize parameters that are functionally important for each application and determine their control values,
2. To maintain accuracy and agreement in engineering surface measurements among various instruments.

The first aspect mentioned above involves controlled experiments. Usually, the parameters and their controlled values, by which the balance between manufacture and function can be well maintained, come from the manufacturer’s experience with the product’s performance and the consumer’s satisfaction. If the balance is not maintained very well, or if either the manufacturing process or the surface function is changed, a controlled experiment should be performed to determine the important surface parameters and their control values, or even the validity of the manufacturing process itself. Otherwise, serious failures could occur when the parts are used. One example comes from an automobile factory line for making camshafts [1]. The manufacturing process there was changed from grinding and lapping to turning and burnishing, but no change was made in the specification of surface texture. Because the texture produced by turning is much rougher than that produced by grinding, the burnishers had to produce an enormous amount of plastic deformation in order to get the cam roughness down to the specified value. This deformation ruined the surface’s ability to withstand stress, which caused the cams to fail in large numbers of cars.

During the controlled experiments, previously specified parameters may be replaced by new parameters, which relate better to specific functional properties of the surface rather than merely statistical parameters characterizing geometrical features. One example is the family of *R*_k_ parameters [34, 35]. The reduced peak height *R*_pk_ serves to describe profile characteristics relating to the running-in behavior of surfaces, the core roughness depth *R*_k_ relates to the long-term running behavior, and the reduced valley depth *R*_vk_ relates to lubricant behavior of the surfaces in static contact.

It is important to measure a large number of surfaces during the controlled experiments in order to ensure that the findings are significant. It is also important to maintain a high degree of repeatability and accuracy in these measurements, which could be very different from that of surface roughness specimen calibrations.

This brings us to the second aspect of engineering surface quality control, the accuracy of engineering surface measurements. Maintaining accuracy involves considerations for: 1) measuring instruments; 2) measured surfaces, including both calibration specimens and the real engineering surfaces; and 3) calibration and measurement procedures. We discuss each consideration in turn below.

#### 1) Measuring Instruments

In a measuring instrument, information about the surface may pass through several stages. In stylus instruments, for example, as the information flows from the stylus tip to the indication on the recorder, the surface information is subject to distortion. Some of this distortion is intentional, such as that produced by profile filters. Other distortion is unavoidable due to practical considerations such as the finite size of the stylus [5]. Therefore, any measurement of a single surface could yield, to a greater or lesser extent, an arbitrary result if there are no limitations on the reference conditions of these measurements.

Different instruments may be manufactured according to different national and international standards whose interrelationships are obscure [6]. Furthermore, many aspects of digital instruments are not covered by national and international standards, and this has led to different instrument philosophies being implemented by the manufacturers. This is most evident when measuring standards or reference specimens. Unless a surface measuring instrument has the same specification as the instrument used during the original calibration and certification of a specimen, the measured parameter value may differ from the marked value. At present the specifications for some aspects of the calibrating instrument are arbitrary [5].

For surface texture results to have any “absolute” interpretation, the standard reference measuring conditions of the measuring instrument must be precisely defined. Thus, the concept of the “reference surface metrology instrument” (RSMI) was suggested [5]. Without a reference instrument conforming to standard reference measuring conditions, direct comparison of parameter values between different instruments is often not satisfactory. And if these disagreements happen for the surfaces of calibration specimens, they can also happen for real engineering surfaces where the measurement problems may be harder to diagnose.

Different calibration laboratories do use different surface measuring instruments. The specification and subsequent adoption of the standard reference measuring conditions by calibration laboratories would ensure compatibility of parameter values of reference specimens, as well as real engineering surfaces.

#### 2) Measured Surfaces — Calibration Specimens and Engineering Surfaces

One of the most interesting parts of instrument development has been that of proving performance. For example, the measurement of small stylus displacements combined with low operating forces and high magnifications required the development of more sensitive proving techniques than have been previously available. Generally the instruments could be adapted to prove themselves [3]. That is, a certified standard could be measured by the instrument to be proved, and the difference between the measured result and the certified value of the standard would show the performance of the instrument. Therefore, the calibration of the existing range of stylus instruments with their wide range of performance calls for different types of calibration specimens. Each type of calibration specimen, with its specially designed surface profile, high uniformity over its surface, and certified parameter values, should be useful for calibrating certain aspects of instrument performance and capable of avoiding a bad calibration due to effects caused by other performance characteristics of the instrument. For example, by using a sinusoidal specimen [36] with small slope and with wavelength long as compared with the stylus size but short when compared with the instrument cut-off length [37], we can calibrate both the vertical and horizontal magnification, or check the *R*_a_ readings of stylus instruments. These calibrations would be insensitive to the effects of stylus tip size or filter cut-off of the calibrated instrument.

Since real engineering surfaces are different from those of most existing calibration specimens, a check specimen with engineering surface features is suggested for use in engineering surface measurement. Its measuring area should also have high uniformity, and this is aided by having a unidirectional profile. The surface waveform, wavelength and roughness parameter values should be comparable to those of the measured engineering surface. The check specimen could be measured under standardized reference measuring conditions, and receive its certified parameter value, or even a set of certified values corresponding to various measurement conditions, such as stylus tip size and filter cutoff length. By comparing the measured result with the certified value, the check specimen thus provides an overall check of proper instrument usage in engineering surface measurement.

#### 3) Calibration and Measurement Procedures

The calibration and measurement procedure involves the use of various calibration and check standards (“specimens”). Each calibrated specimen may have a limited range of application according to its own characteristics and those of the instrument to be calibrated. The validity of the calibration of an instrument would depend on the correct association of these individual characteristics [37].

It should be noted that certain calibration or measurement procedures correspond to the measurement of certain kinds of measured surfaces. At national laboratories, such as ours, the routine surface measurements are made mainly on the calibration specimens. Sometimes, when we measure real engineering surfaces, mostly for research purposes, a larger uncertainty is obtained in our measurements because the positional fluctuation of the engineering surface is appreciably greater than the instrumental uncertainty of an individual measurement [3]. This is an important factor inhibiting researchers from investigating their measuring errors in real engineering surface measurements. However, we believe that the use of a greater number of measurements (larger statistical samples) on these types of surfaces together with improved calibration procedures would significantly enhance the accuracy and usefulness of the surface measurements.

At industrial metrology laboratories, routine surface measurements are performed mostly on the real engineering surfaces. Some of these labs maintain their traceability of surface measurement to NIST by sending their calibration specimens to our lab for periodic calibration and by using the same procedure to measure their engineering surfaces as we did on the specimens. The validity of this traceability depends both on the difference between the calibrated specimens and the measured engineering surfaces, and on the difference between their instrument and ours.

All of these aspects mentioned above affect each other. For example, when a controlled experiment shows that new surface parameters, such as the *R*_k_ family, should be selected for some kind of functional surface quality control, instruments should be enhanced for the measurement of R_k_ parameters. In addition, new calibrated specimens, having highly uniform *R*_pk_, *R*_k_, and *R*_vk_ values close to those of the controlled engineering surfaces, should also be developed as intermediate check specimens relating the reference instrument and the instrument used for engineering surface quality control.

At the NIST surface calibration laboratory, we calibrate scores of reference specimens each year. We also issue the sinusoidal calibration specimens: SRMs (Standard Reference Materials) 2071–2075. In addition, specimens developed elsewhere are often sent here for testing their properties. In this paper, we describe the characteristics, utilizations, and limitations of different classes of precision roughness calibration specimens. We also discuss the effects of various reference conditions on specimen calibration and propose a measurement procedure, which involves the use of a check specimen, for engineering surface quality control. For one class of specimens the surface texture design is intended to meet the functional requirements in engineering surface quality control.

## 2. Calibration and Check Specimens

The reference conditions which should be defined, calibrated, or checked in a stylus instrument measurement are:

- magnification, both in the vertical and horizontal direction;
- the stylus tip;
- the stylus loading;
- the type of skid or reference datum;
- the type of filter, reference line, and cut-off length;
- profile digitization;
- the algorithms for calculating parameters;
- the number and distribution of profiles on the surface.

There are four types of calibration specimens according to the ISO 5436 standard [37], each of them are specially designed for the purpose of calibrating *certain* of the above characteristics of the stylus instrument. It is important to prevent these calibrations from errors caused by the *other* characteristics of the calibrated instrument or by the reference conditions during these calibrations. The four types of calibration specimens are discussed below.

### 2.1 Specimens for the Calibration of Vertical Magnification

At the NIST surface calibration laboratory, a set of step height standards with calibrated step heights ranging from 0.0291 to 22.90 μm is used for the calibration of our stylus instruments. The calibration profiles are taken under unfiltered conditions, and the width of the step or groove on the specimen is wide enough so that the step height measurements are not sensitive to the stylus size. Therefore, our consideration of the reference conditions is focussed on the datum of the step and the algorithm of the step height.

**Figure 1:**
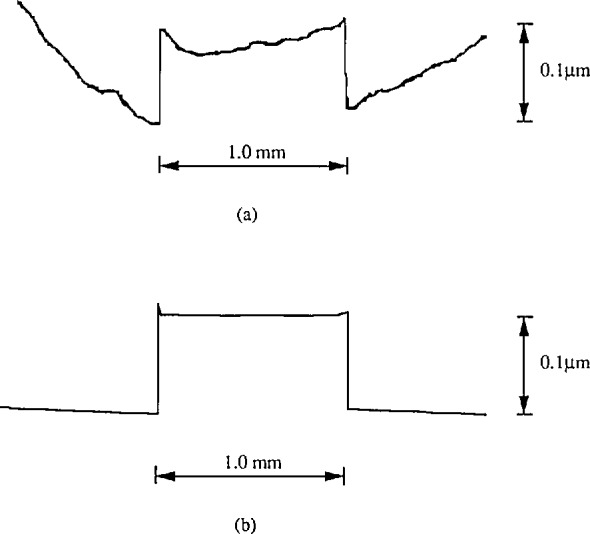
The profile of a step height specimen distorted by the straightness of the traverse mechanism of the stylus instrument. a) skidless; b) with skid.

Most stylus instruments have mechanical reference datums provided by their slides. The straightness of the traverse mechanism along the datum is an important factor. It could be more than 0.1 μm for a 2 mm traversing length. At high vertical magnifications, say 50,000 × or more, such a straightness error can deform the measured step profile seriously (see fig. 1a). The use of the skid can reduce this error to a large extent (see fig. 1b). However, the skid can only be used when the surface has a smooth area which can serve as a datum for the skid to move on. This approach works well as long as the skid is offset sufficiently from the stylus along the direction of travel so that the skid never crosses the step itself but rides only on the outer flat section of the surface. Otherwise, the step could be damaged seriously when the skid touches it.

**Figure 2:**
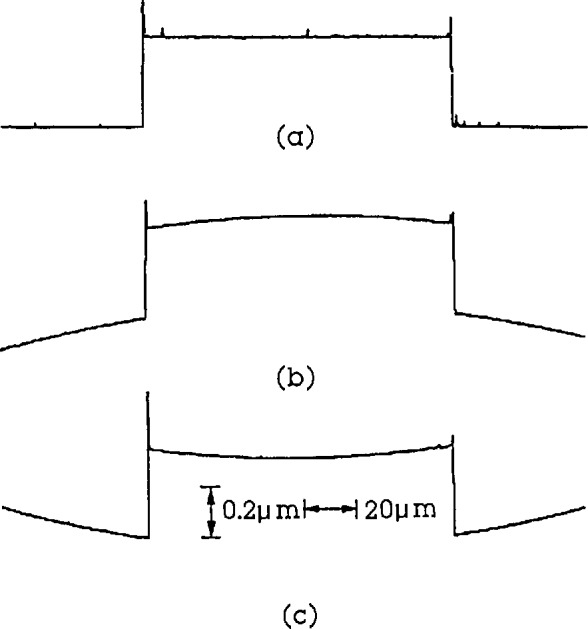
The profile of the step height specimen distorted by the unleveled surface for a stylus instrument with a flexure pivot stage. a) leveled surface; b) and c) unleveled surface.

Flexure pivots are used in some high resolution stylus instruments instead of slides. The resulting path of the stylus is slightly curved in the horizontal plane. If the measured surface is not leveled perpendicular to the general direction of travel, the measured profile of the step could be deformed to be either convex or concave (see fig. 2b, c).

We recently tested the calibration of step height for errors due to curvature in the measured profile. There were two algorithms in the step height calculation. The first was adopted by ISO 5436 [37]. It calculates the step height as the distance between two least square lines as shown in the Appendix. The second one is used at NIST. It calculates the step height as the average of the left and right step heights. Both of these are calculated by one-side step-height algorithms [38]. We now consider the effects on the calculated step heights when the step profile is distorted during the calibration of a stylus instrument or during a step height measurement.

We measured a 0.303 μm two-sided step in leveled and extremely unleveled positions with our high resolution stylus instrument (see fig. 2). In spite of the distortion, we got very repeatable results of 0.3024, 0.3029 and 0.3032 μm corresponding to figure 2a, b, and c, when the step height was determined by the average of the left and right. However, a tremendous error could be obtained corresponding to figure 2b and c if the step height was determined by the two least square lines. This error Δ could be calculated analytically using considerations discussed in the Appendix.

Such extreme profile curvature is unrealistic for a step height measurement. However, a difference of a few percent between results of the two algorithms can arise both in instrument calibration and step height measurement. The step profile can be deformed by the straightness error of the traverse mechanism of the instrument, by an unleveled step surface (for flexure pivot profilers) or by curvature in the specimen itself. These curvature problems become more significant at high magnification. The variations could be remarkably decreased if least squares circular arcs were used instead of least squares lines to determine the step height. That way the algorithm automatically anticipates the surface curvature.

**Figure 3:**
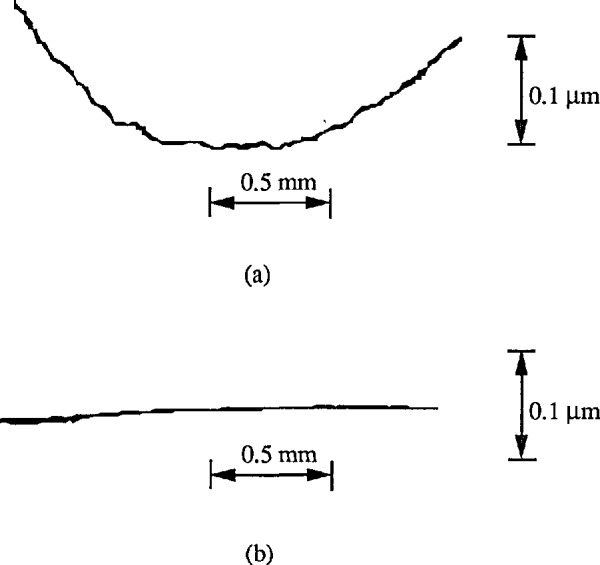
The straightness of the traverse mechanism checked by traversing on an optical flat (lithium niobatc) surface. a) skidless; b) with skid.

The straightness of the traverse mechanism could be checked with the stylus traversing on an optical flat glass surface. For one of our stylus instruments, such a profile segment is shown in figure 3a. The use of a skid could decrease this error to a large extent (see fig. 3b).

### 2.2 Specimens for Checking Stylus Tip Condition

The stylus radius and apex angle have been adopted by most national and international standards [27,39]. However, we have gradually found that the stylus radius is a difficult quantity to define, especially for some chisel-shaped styli [40, 41]. We now prefer stylus width *w*, which is defined to be the distance between the two points of contact when the stylus profile is inscribed in a 150° angle. If the stylus tip has a perfectly round shape, then *w* =0.52*r*[40].

Although several methods, such as SEM, optical microscopy and the razor blade trace, have been developed to measure the stylus size under laboratory conditions [41], the most difficult part of the roughness instrument to check in the workshop is still the stylus [3].

One method involves traversing a calibrated roughness specimen having a series of fine V-shaped grooves, the profiling of which is sensitive to the stylus size. By comparing the roughness value obtained with the true roughness value of the specimen, the stylus size may be determined. These specimens have been widely used for stylus tips over 10 μm. However, finer grooves, useable for checking 2 μm tips, are still difficult to make [3,37].

**Figure 4:**
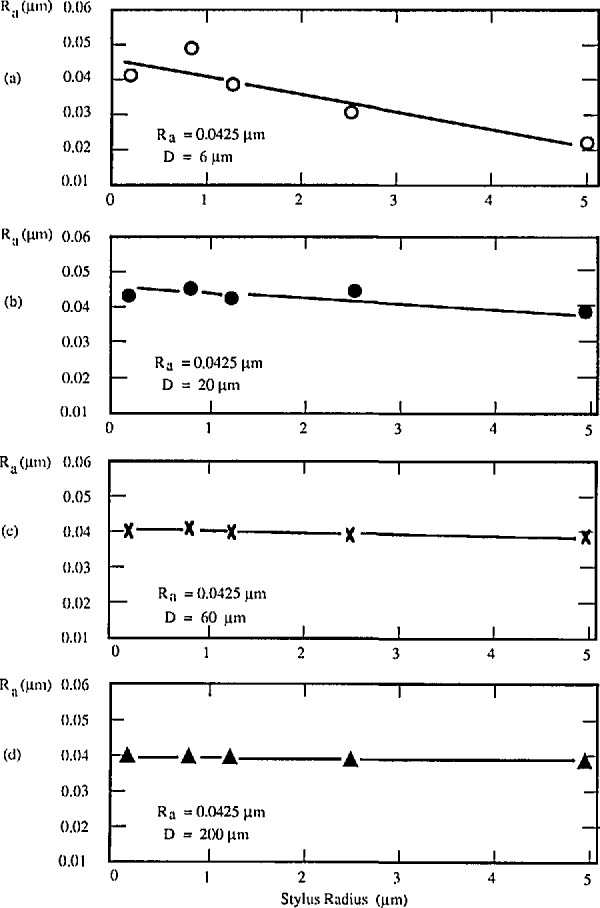
The measured roughness values on a rectangular profile specimen with nominal *R*_a_ = 0.0425 μm and four different pitches [42]. a) *D* = 6 μm; b) *D* = 20 μm; c) *D* = 60 μm; d) *D* = 200 μm.

**Figure 5:**
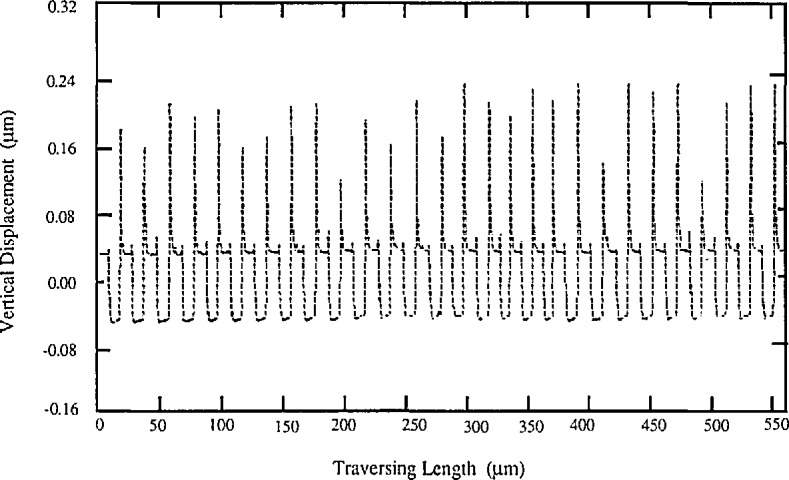
Stylus flight on the surface of a rectangular profile specimen.

One set of specimens is fabricated by oxidation and etching of silicon wafers [42]. Each specimen is composed of square-profile arrays of four distinct pitches, with wavelength 6, 20, 60, and 200 μm. The pitches with 6 and 20 μm wavelength are nearly equal to certain standard stylus sizes and could also be used for testing stylus tips. One of the experiments on such a wafer specimen with nominal *R*_a_ value of 0.0425 μm is shown in figure 4. Roughness average (*R*_a_) values were measured on the four pitches with styli of different nominal radius. The measured *R*_a_ value, which decreases as the stylus size increases, should be less than the nominal value, with the difference depending on the stylus size as well as profile wavelength. Sometimes, when the finer stylus was tested, the measured *R*_a_ value could be larger than the nominal value, because of “stylus flight” (see fig. 5) [43]. The stylus tip was losing mechanical contact with the surface under conditions of very low stylus force. Stylus flight could even happen at a slow traversing speed of 0.122 mm/s on our high resolution stylus instrument.

### 2.3 Periodic Profile Specimens

Various periodic specimens, with rectangular, triangular, arcuate and sinusoidal profiles, are used for the calibration of *R*_a_ readings of stylus instruments. They may also be used for the calibration of horizontal magnification, and the sinusoidal specimens may be used for checking the signal filter characteristics of stylus instruments.

**Figure 6:**
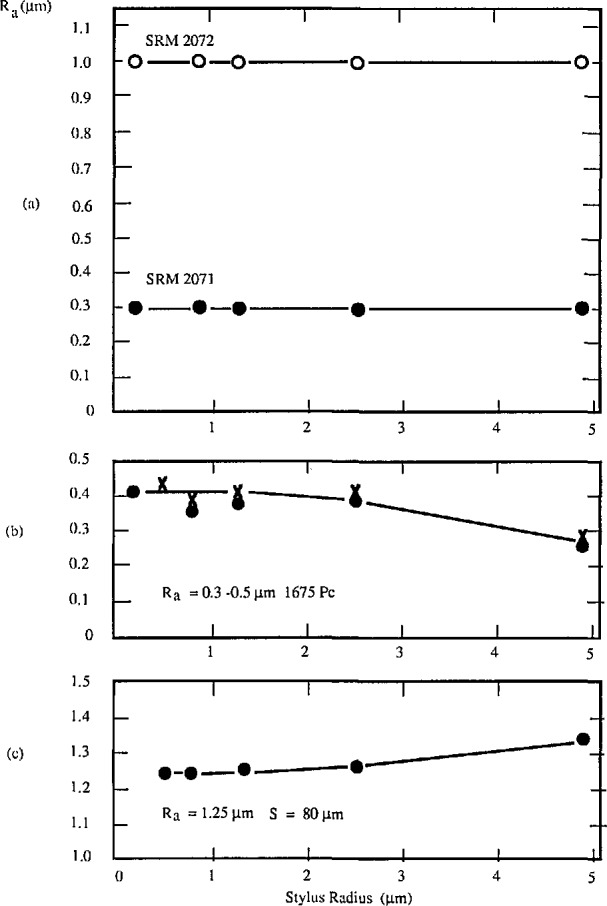
The effects of stylus size on roughness measurements of different periodic profile specimens. The curves are eyeballed fits. a) sinusoidal profile specimens; b) specimens for checking stylus tips; × −*M*_v_=5,000×;·−*M*_v_=20,000× c) profile specimen with cusped peaks.

**Figure 7:**
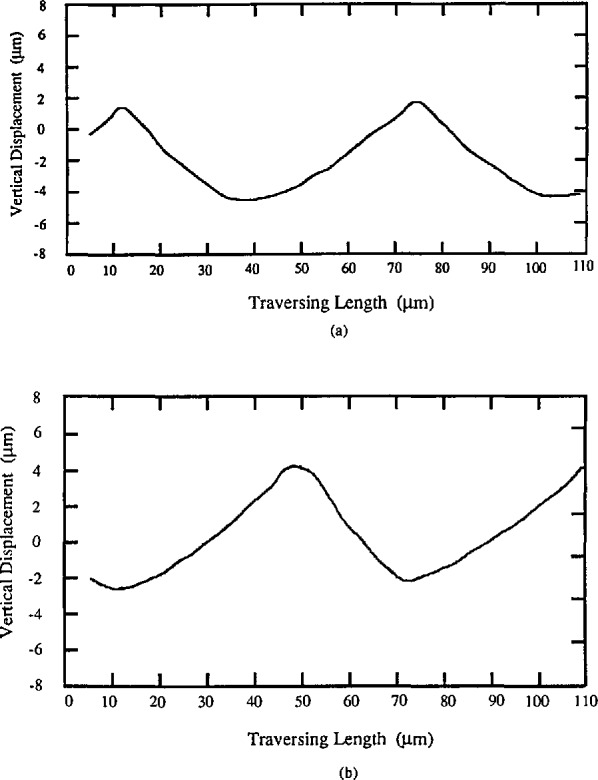
The peaks of the arcuate profile specimen are enlarged by increasing the stylus size. a) stylus width *w* = 0.4 μm, *R*_a_ = 1.26 μm; b) stylus width *w* = 5 μm, *R*_a_ = 1.33 μm.

The wavelength of these periodic specimens should be long enough, say 30 μm or more, that the measured roughness is insensitive to the stylus size; and short enough, say less than 1/10 of the cut-off length, [37] that the measured roughness is insensitive to the filter of the instrument. Figure 6 shows the effect of the stylus size on R_a_ results for different periodic specimens. Results for the sinusoidal specimens with 100 μm wavelength are insensitive (fig. 6a), while those for the triangular specimens with 15 μm wavelength are very sensitive to the stylus size (fig. 6b). As an exception, the *R*_a_ value increases with the stylus size on the specimen with cusped peaks (see fig. 6c). That is because the widths of the peaks were enlarged by the bigger stylus size. Figure 7a shows a profile of the latter specimen measured with a stylus of width 0.4 μm, yielding an accurate profile of the cusped peaks. Figure 7b, on the other hand, was measured with a 5 μm width stylus and therefore shows broadening of the peaks, leading to the increased *R*_a_ value. This effect could even happen on real engineering surfaces with cusped profiles formed by the cutting tool radius during turning, planing, and side milling processes [44].

**Figure 8:**
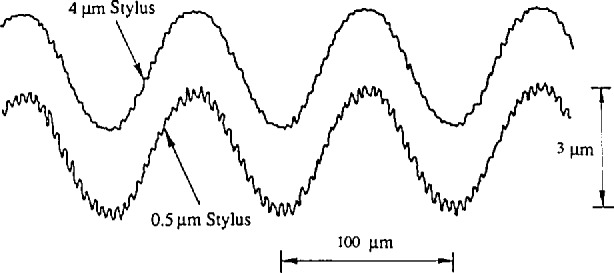
The fine-scale harmonic on the profile of sinusoidal specimen SRM 2072. a) stylus width *W* = 4 μm; b) stylus width *W* = 0.5 μm.

The NIST sinusoidal specimens, SRM 2071–2075, with *R*_a_ values ranging from 0.3 to 3 μm and wavelengths ranging from 40 to 800 μm, were manufactured by the numerical controlled diamond turning process, and calibrated at NIST’s computerized surface calibration system consisting of a stylus instrument integrated with a laser interferometer. The *R*_a_ value was measured by a stylus instrument calibrated by one of our step height calibration specimens. Meanwhile, the surface wavelength was calibrated by using a laser interferometer to measure the lateral displacement of the moving stylus. These sinusoidal specimens can yield constant *R*_a_ values despite different stylus sizes (see fig. 6a). The surface profiles of some of these were machined with a fine-scale harmonic on the sinusoidal profile as shown in figure 8. The spacing of the fine-scale structure was approximately 4.2 μm. It can be useful for monitoring the quality of the fine stylus tips. The upper profile in figure 8 was obtained with a stylus of width 4 μm, while the lower profile was obtained with a stylus of width 0.5 μm. The fine-scale structure can be also useful for checking different zero-crossing and peak-counting algorithms in the surface profile analysis.

**Figure 9:**
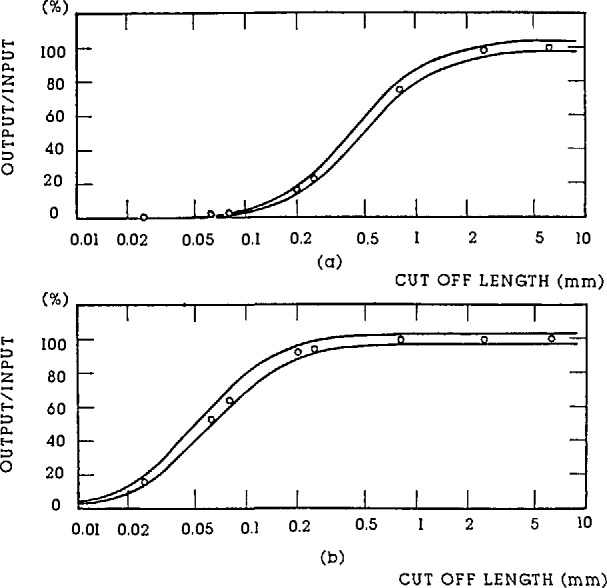
The filter characteristics of the NIST stylus-computerized surface calibration system calibrated by a single sinusoidal specimen. a) by SRM 2075, *R*_a_ = 1 μm, *D* = 800 μm; b) by SRM 2072, *R*_a_ = 1 μm, *D* = 100 μm.

A set of sinusoidal specimens with various amplitudes and wavelengths (λ), λ being equal to 1/3, 1, and 3 × cut-off length, were also suggested in ISO 5436 for the calibration of filter characteristics of stylus instruments [37]. Since our currently existing sinusoidal specimens SRM 2071–2075 cannot cover as wide a range in amplitude and wavelength as discussed in ISO 5436, we can test the filtering characteristics of the instruments at only a few frequencies. We measured the *R*_a_ values of the sinusoidal specimen for various settings of the filter cutoff length, and compared these values with theoretical tolerance values calculated from the B46.1-1985 standard [27] or ISO 3274 [39] corresponding to different cut-off lengths. By this method, we calibrated the filter characteristic of our stylus-computerized surface measuring system as shown in figure 9. All of these measured *R*_a_ values on SRMs 2072 and 2075 for various cutoff lengths of the system are within the tolerance range.

### 2.4 Specimens with Random Profile

Many engineering surfaces have random profiles, such as those manufactured by grinding, lapping, polishing, or honing. Others have profiles with random content superimposed on a periodic profile. These include surfaces manufactured by turning, planing and milling processes. Random profiles have wide amplitude distributions (usually Gaussian) and wide spatial frequency distributions. Measurements of these surfaces with different instruments could produce varying results if the spectral responses of the instruments differ significantly. In 1965, Häsing at PTB [45] developed random profile roughness specimens to represent these types of surfaces. These specimens are made with a measuring area having a unidirectional random profile manufactured by the grinding process. The random profile is repeated every 4 mm, a period exactly equal to the evaluation length of the measurement. Therefore, the measured random profile is always essentially constant within the measuring area, irrespective of the measuring positions on the specimen. These specimens consist of a set of three pieces, with *R*_a_ values of 1.5, 0.5, and 0.15 (or 0.2) μm. With random profile specimens, stylus instruments can be subjected to a summary test covering all stages in the instrument from the stylus tip to the indication of the measured value. These roughness specimens play an important role in getting agreement for engineering surface measurement among various instruments. Hillmann [46] recently reported that a couple of years ago, differences in measured surface parameters of 40% and more were ascertained in national and international comparison measurements. Now, the differences between measurements carried out within the framework of the German Calibration Service amount to only a few percent; in an audit of the European Communities less than 15% was attained [26,46]. Hillmann’s recommendations included the use of both well defined measurement conditions and well characterized roughness specimens.

The PTB roughness standards are limited to *R*_a_ values ≥ 0.15 μm. However, smooth engineering surfaces with *R*_a_ ≤ 0.1 μm play an increasingly important role both in industry and research. Most smooth engineering surfaces, such as those made by fine grinding, lapping, polishing, honing, and electro-polishing, have random profiles. Since the importance of smooth engineering surfaces, as well as their production costs are extremely high, the surface quality control becomes increasingly significant. Stylus and other surface measuring instruments have been designed for this purpose, but their measurement results can be divergent. One of the important reasons is that most smooth engineering surfaces have average spatial wavelengths falling in the range of the stylus size itself (~ 10 μm or less). Therefore, measured results are very sensitive to the stylus size. In addition, their surface texture may vary widely from place to place.

**Figure 10:**
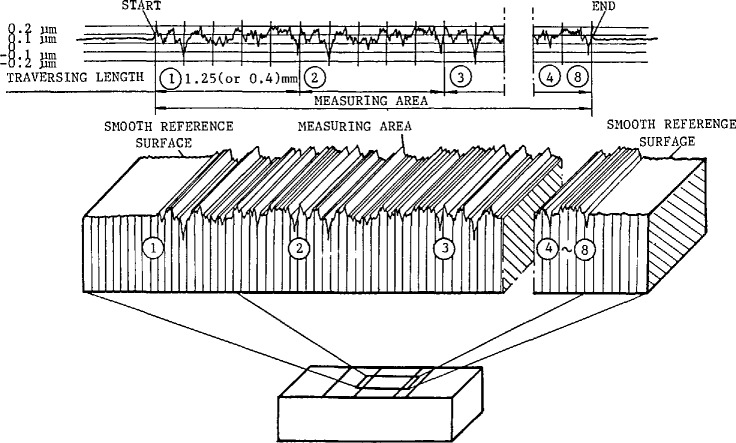
Random profile precision roughness calibration specimen.

In 1986, random profile precision roughness calibration specimens were developed by Song at the ChangCheng Institute of Metrology and Measurement (CIMM) Beijing, China, to aid in identifying various effects in smooth engineering surface measurement [47]. These specimens are similar to PTB random profile roughness specimens but have smoother values of roughness. The measuring areas are composed of several (4–8) identical unidirectional random profile surfaces side by side, and their profile repetitions are equal to the recommended evaluation lengths for measuring them (see fig. 10). The specimens are a set of four pieces having *R*_a_ values of 0.1, 0.05, and 0.025 μm (these three with profile repetition of 1.25 mm) and 0.012 μm (with profile repetition of 0.4 mm).

There are also two smooth reference surfaces at opposite ends of the measuring area (see fig. 10). The smooth reference surfaces were made to be situated on the mean lines of the random roughness profiles and have *R*_a_ ≤ 0.005 μm and flatness ≤ 0.01 μm. The reference surfaces provide a mechanical measuring datum for a skid to move on. By this means, the mechanical noise of the stylus instrument can be reduced to a minimum [47] because the smooth reference datum, rather than being a slide located in the drive box, is located very close to the stylus. This consideration is similar to minimizing the Abbe offset in dimensional measurement. Lines of intersection between the smooth reference surfaces and the measuring area also provide a datum for the start and end positions of the random profiles. It makes possible the comparison between the profile graphs obtained with various instruments.

**Figure 11:**
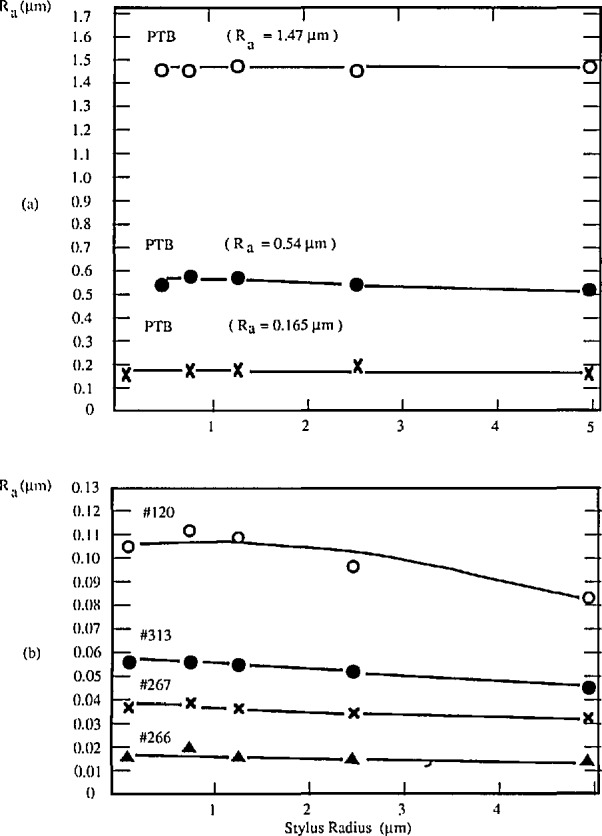
The effects of stylus size of various random profile specimens. a) PTB specimens; b) CIMM specimens.

PTB roughness specimens, with their relatively longer peak spacing do not yield *R*_a_ results that are sensitive to the stylus size [48] (also see fig. 11a). However, CIMM specimens, with mean peak spacing less than 10 μm are very sensitive to the stylus size (see fig. 11b), and could be used for checking stylus condition. To obtain agreement between results on CIMM specimens measured with various instruments, the reference conditions, especially the stylus size, should be carefully adhered to. Like the PTB specimens, the CIMM specimens simulate the situation of measuring real, smooth engineering surfaces and provide a means of exposing the problems or obtaining agreement when stylus and other instruments are used for measuring smooth engineering surfaces.

## 3. Calibration and Measurement Procedures

### 3.1 Coordinating Calibration and Measurement Conditions

**Figure 12:**
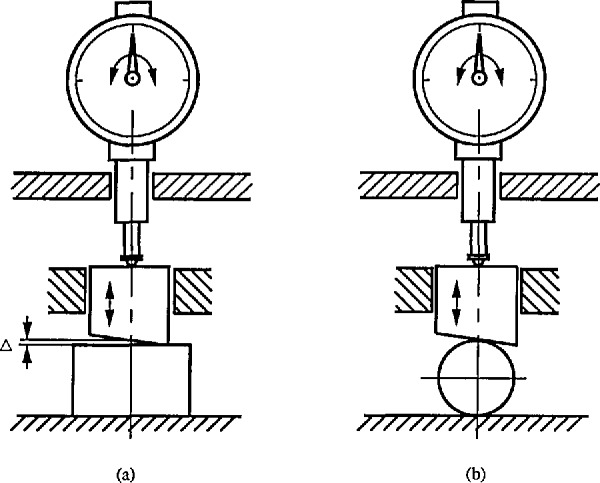
The reference conditions used in the calibration should be as close as possible to those used in the measurement. a) calibration; b) measurement.

One of the important principles in metrology is that the reference conditions in the calibration of the instrument should be as close as possible to those used in the measurement. One example comes from length measurement. Having been calibrated by a calibration block, a height comparator could be used for the measurement of the diameter of a ball (see fig. 12). However, the parallelism between two measuring surfaces has a significant effect on the measuring error. If instead, a certified standard ball with the same size and material as those of the measured balls was used for calibrating the comparator, the error from the parallelism between the two measuring surfaces could be minimized, as well as any error due to the elastic deformation.

**Figure 13:**
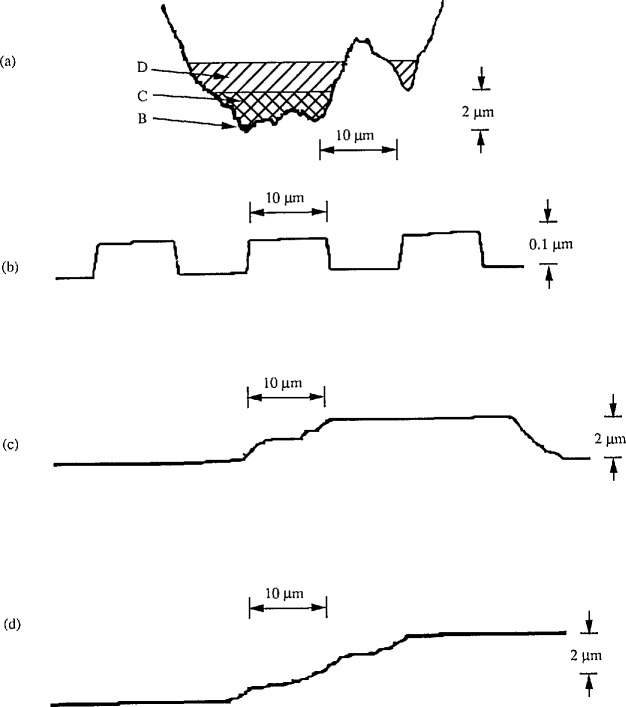
Profiles measured with a defective stylus having a jagged tip profile. a) the profile of the damaged stylus; b) the measured profile of a rectangular roughness specimen, *R*_a_ = 0.0425 μm; *D* =20 μm; c) the measured profile of a rectangular roughness specimen, *R*_a_ = 0.91 μm; *D* = 80 μm; d) the measured profile of a step height specimen, *H* = 3.042 μm.

Another example comes from surface profile measurements. It is well known that a stylus with large width or radius distorts the measured profile seriously and can not be used for measuring the fine structure of smooth surfaces. But certain damaged styli with jagged tips could measure the fine structure of smooth surfaces very well. However, when a rough surface is measured, this type of stylus could cause serious profile distortion. Such a damaged stylus is shown in figure 13a. Using it to measure a wafer specimen [42] with *R*_a_ = 0.0425 μm and *D* =20 μm, we obtained a perfect profile graph (fig. 13b), since only the sharp tip (segment B, fig. 13a) contacted the surface. When we measured a rectangular profile roughness specimen with *R*_a_ = 0.91 μm and *D* =80 μm, we obtained the distorted profile shown in figure 13c because segment C (fig. 13a) of the damaged stylus was involved. The profile was also seriously distorted when measuring a 3.042 μm step height specimen because the damaged stylus profile contacted the surface at several different points in succession. This is shown by segment *D*, figure 13a and d. If only the smoothest rectangular profile had been used to check the stylus tip, we would not have detected the problem of the stylus condition for measuring rougher surfaces. This demonstrates once again the importance of having calibration and checking conditions corresponding to the measurement conditions. Therefore, the calibration or check standards, should be as similar as possible to the measured surface.

### 3.2 Calibration Procedures at NIST

The above principle is being applied in the NIST surface calibration lab. In order to ensure that the calibration or measurement is correct, we measure a check specimen with profiles as similar as possible to the measured surfaces. For example, when calibrating our sinusoidal specimens SRM 2071–2075, we use the following procedure:

- Calibrate the instrument with an interferometerically measured step height specimen, whose height about the same as the amplitude of the specimens to be measured;
- Check the instrument calibration by measuring a certified step height or a certified sinusoidal specimen;
- Measure the specimens under test;
- Check the measurement again by measuring a check specimen as similar as possible to the measured specimens.

Using the check specimen and getting the results within a given tolerance helps to verify the entire set of measurements taken during a run. We also use roughness specimen calibration control charts, in which the measurement results and uncertainty of each calibrated specimen, as well as the calibration constant before and after these calibrations are recorded day by day. The control charts help us to maintain quality control in specimen calibrations and make these calibrations traceable to measurements taken several years ago.

By using the procedure above, we have calibrated sinusoidal specimens, SRM 207-2073. The resulting uncertainties on the *R*_a_ and wavelength values are, for example, (SRM 2072/Serial No. 1021), *R*_a_ = 1.004±0.027 μm (±2.7%) and *D* = 101.64 ±0.24 μm (±0.24%, both ±3σ). Other periodic profile specimens also have waveforms and sufficiently long wavelengths as to be insensitive to the stylus size. For these, we used the same procedure as for the calibration of our sinusoidal specimens, and a sinusoidal specimen with similar *R*_a_ and wavelength was used as a check specimen.

However, our calibrations of some rectangular profile specimens show variations of *R*_a_ with stylus tip size of several percent, even for specimens with a wavelength as long as 80 μm. In these specimen calibrations, we made a detailed report of the reference measuring conditions, especially the stylus size, on the calibration report. If the stylus size used in other labs differ from ours, a few percent difference in the *R*_a_ value could result.

### 3.3 Proposed Procedure for Engineering Surface Measurements

**Figure 14:**
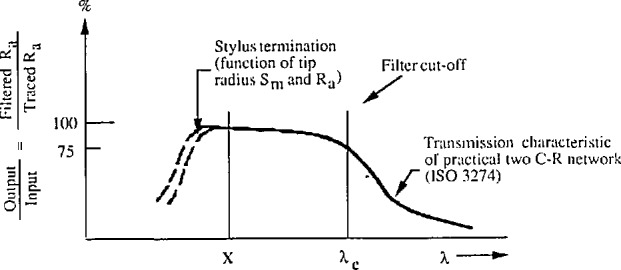
Schematic of the transmission characteristics of the stylus instrument.

If the same procedure were used for engineering surface measurements, one of the important considerations is whether or not the wavelength domain of the measured profile falls in the flat portion of the transmission characteristics of the instrument, as at position X in figure 14 [37], where there is substantially no attenuation caused either by the stylus width at the high end of the spatial frequency spectrum, or by the electrical filter cutoff length λ_c_ at the low end. Otherwise, a big measuring error could occur. As a demonstration, by using the same procedure as that used for our sinusoidal specimens calibration, we measured the same smooth engineering surface with different stylus widths. The measured surface was a CIMM random profile specimen, with the mean spacing of profile irregularities [49] *S*_m_ equal to 6.6 μm. Our results were *R*_a_ = 0.120±0.013 μm and 0.165 ±0.016 μm corresponding to stylus widths of 5 and 0.4 μm, respectively. The two contradictory measuring results should be equally valid, since both of them passed the checking procedure with a sinusoidal specimen as a check specimen which was not sensitive to the stylus size at all. Therefore, if a CIMM random profile specimen were used as a check specimen instead of the sinusoidal specimen, we could spot a change in the stylus condition when measurements of the check specimen are compared with previous ones as on a control chart.

The suggested procedure to be used for engineering surface measurement, as well as in the calibration of reference specimens with a stylus instrument is as follows:

1. Calibrate the vertical magnification of the instrument using a calibrated step height specimen whose height covers the range of the amplitudes of the measured profile;
2. Verify that the calibration was correct by measuring a certified step height, or an *R*_a_ value of a calibrated roughness specimen, such as a sinusoidal specimen;
3. Measure the engineering surface or specimen to be calibrated;
4. Check the measurement by measuring a check specimen with the same or similar waveform to that of the measured surface. The *R*_a_ value of the check specimen should have been measured under standardized reference measuring conditions.

In addition the reference conditions for the stylus instrument measurement, such as filter setting, stylus loading, and straightness of the mechanical motion, should also be carefully checked periodically.

Existing roughness calibration specimens could be used as the check specimens for a wide range of engineering surface measurements. For example, when the measured engineering surfaces have mainly periodic profiles, such as those obtained by turning, planing, or side milling processes, the periodic roughness specimens with triangular, cusped-peak, and sinusoidal profiles could be used as check standards. When the measured engineering surfaces have random profiles, as is obtained by grinding, lapping, polishing and honing processes, the PTB and CIMM random roughness specimens could be used. They would cover the range of *R*_a_ values from 1.5 to 0.012 μm. If the checking measurement shows that the difference between the measured result for the check specimen and its certified value under reference conditions was within a given tolerance, the measurement of the engineering surface is considered to be under good quality control.

At present, different stylus size standards are adopted in different countries, for example, a 10 μm radius in the U.S. [27] and 2 μm radius in ISO and Europe [39,2]. However, there is not a uniformly defined method for checking the effective stylus size in the workshop. Therefore, it is difficult to get agreement in measurements of smooth engineering surfaces. However, the CIMM specimens calibrated by a reference instrument under standardized reference measurement conditions with a fine stylus size could be used as check standards. They make it possible to validate the results of smooth engineering surface measurements and obtain agreement by various instruments.

## 4. Conclusions and Recommendations

1. Engineering surface quality control, which involves the cycle of manufacture, texture measurement, and functional usage of the engineering surface, consists in choosing the right parameters to measure and obtaining knowledge of the right control values, as well as in maintaining the accuracy of the engineering surface measurement.
2. By controlled experiments, the right surface parameters and their controlled values could be determined for each application. If the manufacturing process or the surface function changed, a new controlled experiment should be performed so that a clear relationship between surface manufacture and function could be maintained. During these controlled experiments, new functional parameters, such as the *R*_k_ family, could be adopted.
3. Accuracy of engineering surface measurement would be aided by the establishment of a Reference Surface Metrology Instrument (RSMI), which would verify the standardized reference measuring conditions, and by the use of various calibration and check specimens with their certified parameters measured with the RSMI. A correct measurement procedure would include the choice and use of the check specimen for engineering surface measurement.
4. The check specimen’s waveform, and parameter values should be similar to those of the measured engineering surfaces. The parameter value certified by the RSMI (or under standardized reference measuring conditions), provides an overall checking mechanism for all processes in the instrument including stylus tip, filter, and gain. The measuring results are accepted only when the check specimen is measured with the same instrument and a result within a given tolerance of its certified value is obtained.
5. Some precision roughness calibration specimens could be used as check specimens for a wide range of engineering surface measurement, from periodic to random profile, from several μm to 10 nm *R*_a_ But there are still some new specimens that should be developed. These include specimens for testing stylus conditions, and specimens for testing scanning tunneling microscopes and atomic force microscope to be used in super-smooth surface measurements.
6. With the development of the functional parameters, such as the Frech R & W [50,51] and German *R*_k_ family [34,35], the function of engineering surfaces could be assessed more quantitatively. Therefore, it may be possible to optimize functional performance of engineering surfaces by designing their surface texture, material, manufacturing processes, and quality control procedures. We call this combination the “surface texture design.” For example, during the finishing of engine cylinder bores by the plateau honing process following the honing process [52], an optimal cylinder bore surface texture could be obtained. This surface has a negatively skewed profile height distribution, (negative *R*_sk_) [49] which results in good performances for running-in, long-term running and lubrication of the cylinder bores. The *R*_k_ family of parameters including algorithms and software have been developed for the measurements of such engineering surfaces. Meanwhile, some new specimens should also be developed to simulate these designed surface textures and used as check specimens in engineering surface quality control. These specimens could be manufactured by combining the manufacturing techniques of the PTB and CIMM specimens.
7. Replication techniques make it possible that the “same” engineering surface could be used as a check specimen on a great number of instruments. Various electro-formed replica specimens with periodic profile have been successfully used in engineering surface measurement. However, for random profile roughness calibration specimens, especially those of *R*a ≤ 0.1 μm, the agreement between the original and the replica specimens would have to be carefully investigated so that the comparison among various instrument measurements could be based on the “same” smooth engineering surface.

## Figures and Tables

**Figure 15:**
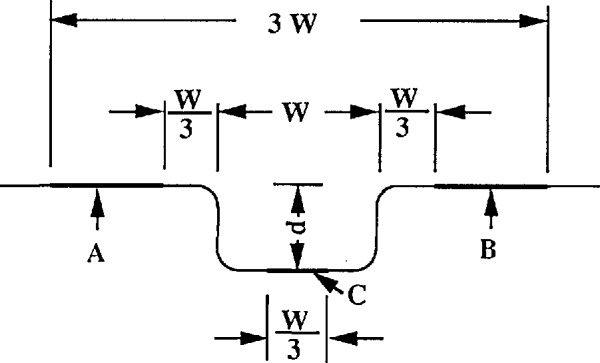
Assessment of step height according to the ISO 5436 Standard.

**Figure 16:**
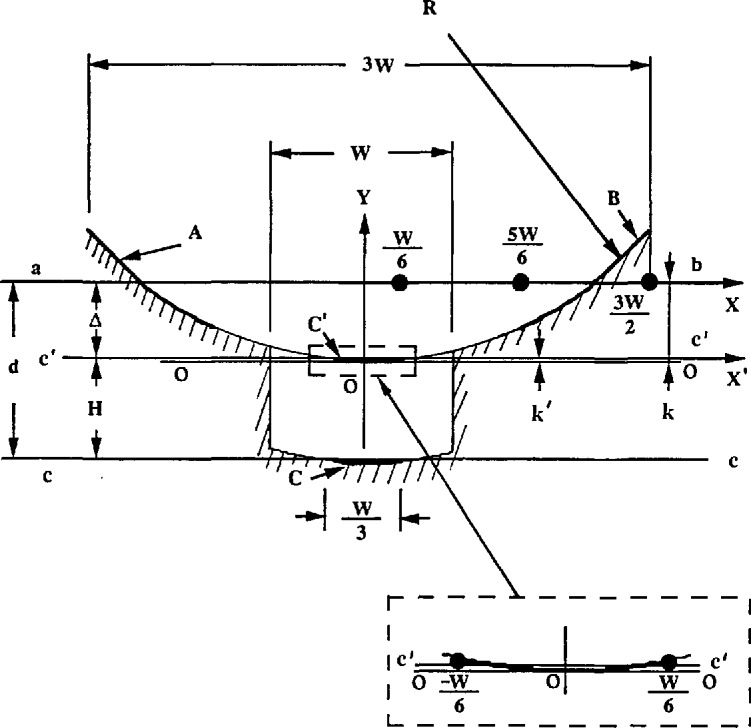
The error of step height when the profile graph is distorted into a curve.

## 5. Appendix

We discuss here certain difficulties with using the ISO Standard 5436 for step height measurement on curved surfaces. According to ISO 5436, the depth of a groove (fig. 15) or the height a step is determined as follows:

“A continuous straight mean line equal in length to three times the width of the groove (3w) is drawn over the groove to represent the upper level of the surface and another (w/3) to represent the lower level, both lines extending symmetrically about the centre of the groove (see fig. 15).

“To avoid the influence of any rounding of the corners, the upper surface on each side of the groove is to be ignored for a length equal to one-third of the width of the groove. The surface at the bottom of the groove is assessed only over the central third of its width. The portions to be used for assessment purposes are therefore those shown at A, B, and C in figure 15.

“The depth *d* of the groove shall be assessed perpendicularly from the upper mean line to the midpoint of the lower mean line.

“*NOTE:* The depth *d* as described here will be equal to the mean of the portion C below the upper mean line.”

We now consider the effect when the profile graph is distorted into a curve, perhaps because of the straightness error of the traverse mechanism of the stylus instrument, or because of surface curvature.

As before, the step height *d* is determined by the distance between two parallel least square lines ab and cc, both of them calculated from the curved profile, parts A, B, and C (fig. 16).

Since *R> >H*, where *H* is the true step height, the error A caused by the distorted profile could be determined by the distance between lines c′c′ and ab, where c′c′ is a least square line calculated from the curve Part C′, which is situated on the same curve with parts A and B, and parallel to the profile part C with a distance *H* (the true step height). Therefore, we have

$$\Delta =d-H=k-k^{\prime }$$

where *k* and *k′* represent the positions of the least square lines ab and c′c′ with respect to the origin 0.

The deformed profile, parts A and B, could be represented by

$$Y=R-{\left(R^{2}-x^{2}\right)}^{1/2}.$$

The least square line $\bar{y}=k$ could be calculated from

$$\begin{array}{l} f\left(x\right)=\int_{5w/6}^{3w/2}{\left(y-\bar{y}\right)}^{2}dx=\min,\\ f\left(x\right)=\int_{5w/6}^{3w/2}[{\left(R-k\right)}^{2}-2\left(R-K\right){\left(R^{2}-x^{2}\right)}^{1/2}\\ +\left(R^{2}-x^{2}\right)]dx\\ =\{{\left(R-k\right)}^{2}x-\left(R-k\right)[x{\left(R^{2}-x^{2}\right)}^{1/2}\\ +R^{2}\;\sin^{-1}\frac{x}{R}]+{R^{2}x-\frac{1}{3}x^{3}\}}_{5w/6}^{3w/2}. \end{array}$$

Since R > >x,

$$\begin{array}{l} f\left(x\right)\approx \{{\left(R-k\right)}^{2}x-\left(R-k\right)\left[Rx\left(1-\frac{x^{2}}{2R^{2}}\right)+Rx\right]\\ +R^{2}x-\frac{1}{3}{x^{3}\}}_{5w/6.}^{3w/2} \end{array}$$

Let

$$\frac{\partial \;f\left(x\right)}{\partial \left(R-k\right)}=0.$$

Then,

$$\begin{array}{l} {\left[2\left(R-k\right)x-2Rx\left(1-\frac{x^{2}}{4R^{2}}\right)\right]}_{5w/6}^{3w/2}=0,\\ \frac{4}{3}w\left(R-k\right)-3\;Rw\left(1-\frac{9w^{2}}{16R^{2}}\right)\\ +\frac{5}{3}Rw\left(1-\frac{25w^{2}}{144R^{2}}\right)=0,\\ \frac{4}{3}w\left(R-k\right)-\frac{4}{3}Rw+\frac{151}{108}\frac{w^{3}}{R}=0,\\ R-k=R-\frac{151}{144}\frac{w^{2}}{R},\\ k=\frac{151}{144}\frac{w^{2}}{R}. \end{array}$$

The deformed profile Part C′ could also be determined by

$$y\prime =R-{\left(R^{2}-x\prime^{2}\right)}^{1/2}.$$

The second least square line ${\bar{y}}^{\prime }=k^{\prime }$ could be calculated from

$$f\prime \left(x\prime \right)\int_{o}^{w/6}\left(y\prime -\bar{y}\prime \right)dx=\min.$$

Let

$$\frac{\delta f\prime \left(x\right)}{\delta \left(R-k\prime \right)}=0.$$

Then

$$\begin{array}{l} {\left[2\left(R-k\prime \right)x-2Rx\left(1-\frac{x^{2}}{4R^{2}}\right)\right]}_{o}^{w/6}=0.\\ k\prime =\frac{1}{144}\frac{w^{2}}{R}. \end{array}$$

Therefore,

$$\Delta =k-k\prime =\frac{\left(151-1\right)}{144}\frac{w^{2}}{R}=\frac{25}{24}\frac{w^{2}}{R}\approx 1.04\frac{w^{2}}{R}.$$

Since *R* > > x, the radius *R* could be calculated by observing the depth or height (*y*) of the curvature over the entire profile and using the formula below:

$$R\approx \frac{x^{2}}{2y}.$$

From figure 2b, *y* is calculated by construction to be 0.136 μm. We therefore calculate *R* =3676 mm. As a result

$$\Delta =1.04\frac{w^{2}}{R}=0.283\;\mu m\;\left(+93\%\right).$$

A similar result is obtained for figure 2c.
